# Saturation Mapping of a Major Effect QTL for Stripe Rust Resistance on Wheat Chromosome 2B in Cultivar Napo 63 Using SNP Genotyping Arrays

**DOI:** 10.3389/fpls.2017.00653

**Published:** 2017-04-26

**Authors:** Jianhui Wu, Qilin Wang, Shengjie Liu, Shuo Huang, Jingmei Mu, Qingdong Zeng, Lili Huang, Dejun Han, Zhensheng Kang

**Affiliations:** ^1^State Key Laboratory of Crop Stress Biology for Arid Areas, College of Plant Protection, Northwest A&F University Yangling, China; ^2^State Key Laboratory of Crop Stress Biology for Arid Areas, College of Agronomy, Northwest A&F University Yangling, China

**Keywords:** adult-plant resistance, bulked segregant analysis, molecular markers, *Puccinia striiformis*, Triticum aestivum

## Abstract

Stripe rust or yellow rust (YR), caused by *Puccinia striiformis* f. sp. *tritici* (*Pst*), is one of the most important diseases of wheat (*Triticum aestivum* L.). Widespread deployment of resistant cultivars is the best means of achieving durable disease control. The red grain, spring wheat cultivar Napo 63 produced by CIMMYT in the 1960s shows a high level of adult-plant resistance to stripe rust in the field. To elucidate the genetic basis of resistance in this cultivar we evaluated 224 F_2:3_ lines and 175 F_2:6_ recombinant inbred lines (RILs) derived from a cross between Napo 63 and the *Pst*-susceptible line Avocet S. The maximum disease severity (MDS) data of F_2:3_ lines and the relative area under the disease progress curve (rAUDPC) data of RILs were collected during the 2014–2015 and 2015–2016 wheat growing seasons, respectively. Combined bulked segregant analysis and 90K single nucleotide polymorphism (SNP) arrays placed 275 of 511 polymorphic SNPs on chromosome 2B. Sixty four KASP markers selected from the 275 SNPs and 76 SSR markers on 2B were used to identify a chromosome region associated with rust response. A major effect QTL, named *Qyrnap.nwafu-2BS*, was identified by inclusive composite interval mapping and was preliminarily mapped to a 5.46 cM interval flanked by KASP markers *90K-AN34* and *90K-AN36* in chromosome 2BS. Fourteen KASP markers more closely linked to the locus were developed following a 660K SNP array analysis. The QTL region was finally narrowed to a 0.9 cM interval flanked by KASP markers *660K-AN21* and *660K-AN57* in bin region 2BS-1-0.53. The resistance of Napo 63 was stable across all environments, and as a QTL, explained an average 66.1% of the phenotypic variance in MDS of F_2:3_ lines and 55.7% of the phenotypic variance in rAUDPC of F_5:6_ RILs. The short genetic interval and flanking KASP markers developed in the study will facilitate marker-assisted selection, gene pyramiding, and eventual positional cloning of *Qyrnap.nwafu-2BS.*

## Introduction

Global wheat production is affected by many diseases, among which the rusts are the most important (McIntosh et al., 1995; Hovmøller et al., 2010). Stripe or yellow rust (YR) caused by *Puccinia striiformis* f. sp. *tritici* (*Pst*) is a constant threat and as one of the most destructive diseases, it is capable of causing 5–25% yield losses, or sometimes more, in almost all wheat-growing regions (Chen, 2005; Wellings, 2011). Major stripe rust epidemics have occurred in China, with losses in some instances amounting to several million metric tons (Li and Zeng, 2002; Wan et al., 2007; Chen et al., 2009; Kang et al., 2010; Zhao et al., 2016). The most sustainable control strategy is resistant commercial cultivars (McIntosh et al., 1995; Li and Zeng, 2002; Wiesnerhanks and Nelson, 2016).

Stripe rust resistance is usually categorized as all-stage resistance (ASR) and adult-plant resistance (APR) (Chen, 2005, 2013). All-stage resistance is expressed during all plant growth stages and can be detected in seedlings. ASR is often preferred by breeders because the individual genes confer high levels of resistance and are easily selected in breeding. However this type of resistance is more vulnerable to pathogen evolution when deployed singly in cultivars (Chen, 2005; Chen et al., 2010). In contrast, APR, which is characterized by variable levels of response determined by actual growth stage and temperature, usually provides protection against a broader range of races and tends to be more durable (Chen, 2013; Brown, 2015; Niks et al., 2015). Acceptable levels of APR are often due to the interactive effects of two or more genes. The best known *Pst*-APR genes *Yr18, Yr36*, and *Yr46* that confer a degree of resistance to multiple races have been cloned, thus permitting development of ‘perfect’ markers for marker-assisted breeding (Fu et al., 2009; Krattinger et al., 2009, 2013; Forrest et al., 2014; Moore et al., 2015), and most importantly these genes have shown durability. Therefore, combining APR genes with race specific ASR genes is a preferred strategy for wheat breeding as it may prolong the life of the ASR genes and will significantly reduce losses if virulent races do develop (Chen, 2013; Ellis et al., 2014). This highlights the significance of identifying new resistance genes, especially those effective against a broad spectrum of pathogen races.

Quantitative trait loci (QTL) mapping is the currently preferred method of dissecting the genetic components of disease resistance (Niks et al., 2015; Wiesnerhanks and Nelson, 2016). However, the biggest bottleneck for conventional QTL mapping in bread wheat is lack of high-density polymorphic markers; consequently target QTL are often located across a large region and markers may not be sufficiently accurate for map-based cloning of candidate genes or for marker-assisted selection (MAS) in crop breeding (St Clair, 2010; Yang et al., 2015). Recent progress in genome sequencing and high throughput single nucleotide polymorphism (SNP)-based genotyping technologies in wheat has facilitated faster development of trait-linked markers (Yang et al., 2015). Compared with earlier markers, SNP have a distinct advantage in abundance and polymorphism. Recent applications of next-generation sequencing (NGS) greatly improved the efficiency and throughput of SNP discovery that contributed to the use of assay platforms (Wang et al., 2015). Current SNP assay platforms, including Illumina Bead Array^TM^, Affymetrix Gene Chip^TM^ and Kompetitive allele-specific PCR (KASP^TM^),^1^ have been rapidly adopted in mapping and MAS studies (Barabaschi et al., 2016; Rasheed et al., 2016). Moreover, the recently developed high-density 9, 35, 90, 660, and 820K arrays for wheat are efficient for genetic mapping and exceed precious resources in fine mapping (Allen et al., 2013, 2016; Cavanagh et al., 2013; Wang et al., 2014; Jia and Zhao, 2016; Winfield et al., 2016). A lot of research on genome-wide association (GWAS) and QTL mapping of resistance to stripe rust by wheat SNP arrays has already been carried out (Zegeye et al., 2014; Hou et al., 2015; Liu et al., 2015, 2016; Maccaferri et al., 2015b).

Bulked segregant analysis (BSA) (Michelmore et al., 1991), involving selected and pooled DNA samples from contrasting phenotypic groups, provides a simple and rapid approach to search for markers linked to specific genomic regions associated with a trait of interest. Combining the BSA strategy with a high-throughput NGS technology is a common practice for gene identification and QTL mapping. Many studies have outlined methodologies and applications of high-throughput sequencing in BSA for qualitative and quantitative traits (Abe et al., 2012; Takagi et al., 2013; Win et al., 2016).

In a previous study we screened a diverse panel of over 1,000 wheat germplasms for resistance to stripe rust in field nurseries at Yangling in Shaanxi province, and Tianshui in Gansu province. We identified a number of common wheat genotypes with resistance to prevalent Chinese *Pst* races (Han et al., 2012). Napo 63 with the pedigree Frocor//Frontana/Yaqui 48/3/Narino 59^2^ is a red grained spring wheat cultivar produced by the International Maize and Wheat Improvement Center (CIMMYT) in the 1960s. Here, we identify Napo 63 as possessing typical APR to stripe rust. The objectives of the study were to (1) fine map the major QTL for stripe rust resistance using SNP arrays following BSA, (2) identify the candidate resistance genes, and (3) develop and verify the applicability of KASP markers to enable MAS in wheat breeding programs.

## Materials and Methods

### Plant Materials

The mapping population comprised 175 F_5:6_ recombinant inbred lines (RILs) developed by single-seed descent from the cross Avocet S × Napo 63. A second population of 222 F_2:3_ lines from the same cross was derived from a single F_1_ plant. Avocet S is a highly susceptible spring wheat selection from Australia. The wheat cultivars (or lines) Avocet S, Mingxian 169 (MX169) and Xiaoyan 22 (XY22) were used as susceptible controls throughout the study. Two hundred and fifteen wheat entries were used to evaluate polymorphisms of molecular markers flanking the resistance locus. They included 37 leading cultivars, 65 advanced lines from the major winter wheat growing areas in China (Zeng et al., 2014), 28 landraces and 85 foreign germplasms with resistance to stripe rust (Han et al., 2012).

### Greenhouse Trials

Seedling and adult-plant tests were conducted under controlled greenhouse conditions to characterize the APR of Napo 63. Five races (CYR31, CYR32, CYR33, V26/CH42, and V26/Gui22-9) were used. Their virulence/avirulence characteristics were previously reported by Wu et al. (2016). For seedling tests 10–15 plants of Avocet S and Napo 63 were grown in 9 cm × 9 cm × 9 cm pots, and for adult-plant tests three plants were grown in larger 20 cm × 20 cm × 15 cm pots. Seedlings at the two-leaf stage (14 days after planting) and adult-plants at the booting stage were separately inoculated with urediniospores of each race mixed with talc (approximately 1:20). Inoculated plants were incubated at 10°C in a dew chamber in darkness for 24 h, and then transferred to a greenhouse at 17 ± 2°C with 14 h of light (22,000 lx) daily. Infection types (ITs) were recorded 18–21 days after inoculation using a 0–9 scale (Line and Qayoum, 1992). Plants with ITs 0–6 were considered resistant, and plants with ITs 7–9 were considered susceptible. In order to confirm and clarify ITs of the entries, the tests were repeated at three different times.

### Field Trials

Field trials were conducted in five environments at three locations (Yangling in Shaanxi, Tianshui in Gansu, and Jiangyou in Sichuan) in two winter wheat seasons (2014–2015 and 2015–2016), with each year location being considered a single environment. The 175 Avocet S × Napo 63 F_5:6_ RILs were tested at all three locations during 2015–2016 and the 222 F_2:3_ lines were tested at Yangling and Tianshui during 2014–2015. All trials were arranged in randomized complete blocks with three replicates. An individual plot consisted of a single 1 m row with 30 cm between adjacent rows. Each plot was sown with approximately 30 seeds of each RIL or parent, whereas each F_2:3_ line and parents were grown from 25 seeds. The parents and susceptible variety XY22 were planted after every 20 rows throughout the field. Another susceptible control MX169 was planted in 5-row blocks after every 50 rows and around the field area. XY22 and MX169 served as inoculum spreaders to ensure uniform stripe rust development throughout the field. Since, there is reliable natural occurrence of stripe rust at Tianshui and Jiangyou the experiments were conducted under naturally infected conditions. The Yangling site was inoculated with urediniospores of race CYR32 suspended in liquid paraffin sprayed onto MX169 at flag leaf emergence. Disease severities (DS) were recorded three times for each line, when MX169 had 60–100% and XY22 had 30 to >90% severity during the period April 1 to 15 at Jiangyou, May 1 to 20 at Yangling, and May 25 to June 15 at Tianshui. DS were assessed visually using percentage diseased leaf area based on the modified Cobb scale (Peterson et al., 1948). DS for the F_6_ RIL population were converted to area under the disease progress curve (AUDPC) values following Chen and Line (1995). Relative AUDPC (rAUDPC) values were calculated for each RIL and parent as a percentage of the mean AUDPC value of the susceptible parent, Avocet S (Lin and Chen, 2007). Maximum disease severities (MDS) of F_2:3_ lines were scored when YR levels on Mingxian 169 and Avocet S reached their maxima around 15–20 May at Yangling and 10–15 June at Tianshui. Non-segregating lines were scored as a single value; segregating lines were scored as two or more values that were later converted to an average value for analysis.

### Statistical Analyses

Mean MDS of each F_2:3_ line and rAUDPC of each F_6_ RIL were used in analyses of variance (ANOVA). Analyses of variance and Pearson’s correlation coefficients were performed with AOV functionality in the QTL IciMapping V4.1 software package (Wang, 2009) ^3^. Broad-sense heritability ($h_{b}^{2}$) of stripe rust resistance was calculated by $h_{b}^{2}$= $\sigma_{g}^{2}$/($\sigma_{g}^{2}$+ $\sigma_{\mathrm{ge}}^{2}$/e +$\sigma_{𝜀}^{2}$*re*) (Allard, 1960), where $\sigma_{g}^{2}$ is (*MS*_f_ – *MS*_fe_)/re, $\sigma_{\mathrm{ge}}^{2}$ is (*MS*_fe_ – *MS*_e_)/r and $\sigma_{𝜀}^{2}$ is *MS*_e_; in this formula, *σ2 g* = genetic variance, $\sigma_{\mathrm{ge}}^{2}$= genotype × environment interaction variance, $\sigma_{𝜀}^{2}$= error variance, *MS*_f_ = mean square of genotypes, *MS*_fe_ = mean square of genotype × environment interaction, *MS*_e_ = mean square of error, r = number of replications, and e = number of environments.

### Combined Bulk Segregant Analysis with the 90 and 660K SNP Arrays

**FIGURE 1:**
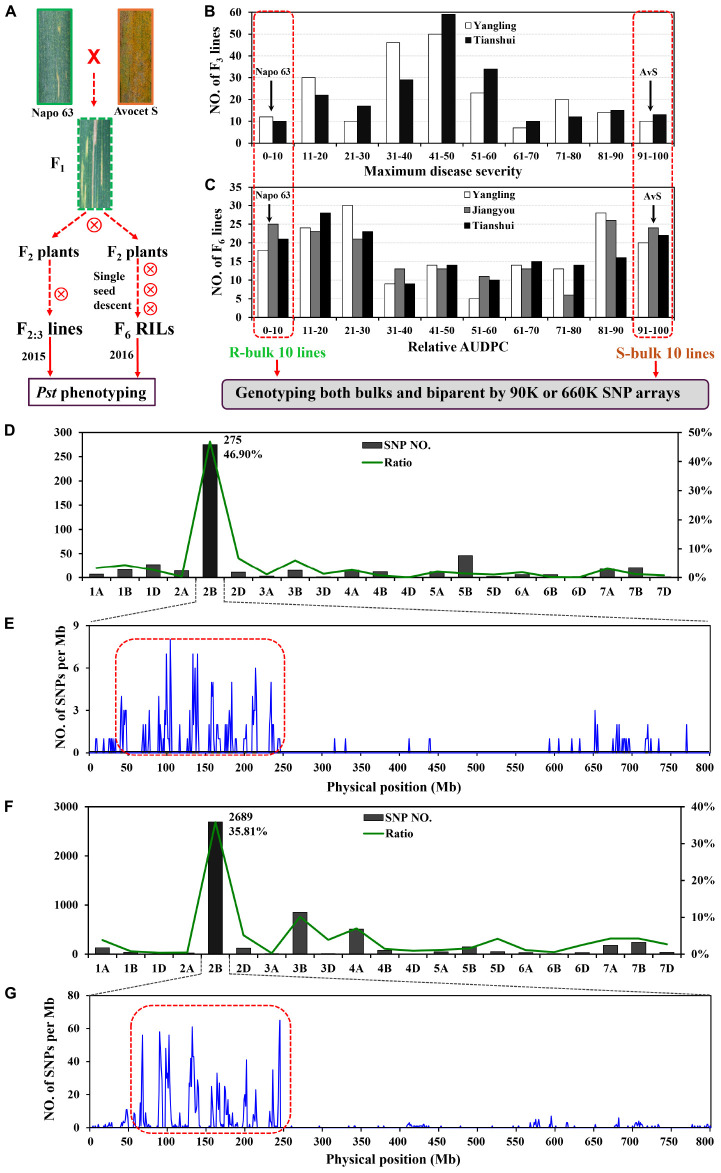
**Overview of analyses.** F_2:3_ lines and F_5:6_ recombinant inbred lines (RILs) were derived from the cross AvS × Napo 63. **(A)** Phenotypes of AvS, Napo 63 and their progenies across all environments and data collected at heading-flowering stage. **(B)** Frequency distribution of maximum disease severity (MDS) for 221 F_2:3_ lines grown at Yangling and Tianshui in 2015. **(C)** Frequency distribution of relative area under the disease progress curve (rAUDPC) for 175 F_6_ RILs grown at at Yangling, Jiangyou and Tianshui in 2016. Black arrows indicate the parental line means. Distributions of the polymorphic SNPs in each chromosome by 90K **(D)** and 660K **(F)** SNP arrays and positions of SNPs in chromosome 2B **(E,G)**. Selected SNPs (in the red dotted boxes) were analyzed in KASP assays.

DNA from parents, F_2_ plants and F_5_-derived lines were extracted from young leaves of greenhouse-grown plants following Song et al. (1994). BSA was performed to identify markers polymorphic between the resistant parent Napo 63 and susceptible parent Avocet S, and between the resistant DNA (R-bulk) and susceptible DNA (S-bulk) bulks. DNA of 10 F_2:3_ lines and 10 F_5:6_ RIL homozygous resistant (IT 1, DS ≤ 5) and homozygous susceptible (IT 9, DS ≥ 90) in all environments were separately pooled to constitute contrasting bulks that along with the parents were genotyped with the 90 and 660K SNP arrays from CapitalBio Corporation (Beijing, China^4^) (**Figures 1A–C**). SNP genotype calling and clustering was performed with Genome Studio Polyploid Clustering v1.0 software (Illumina^5^). SNP markers that were monomorphic, poor quality showing more than 20% missing values, ambiguous in calling, or with minor allele frequencies below 5% were not used. Polymorphic SNPs associated with resistance in BSA were localized to chromosomes based on the high-density 90K (Wang et al., 2014) or 660K genetic maps (Jizeng Jia, personal communications). The sequences of all SNP were used to blast version 0.4 of the assembled Chinese Spring survey sequence [The International Wheat Genome Consortium (IWGSC)^6^] to determine their physical positions.

### Molecular Marker Analysis and Genotyping

Following chromosome location based on polymorphic SNPs, the parents and contrasting DNA bulks were used to screen published wheat SSR markers on that chromosome (Somers et al., 2004). The SSR assays were performed in a S1000 Thermal Cycler (BIO-RAD) with reaction mixtures (15 μL) containing 50–100 ng of template DNA, 1.0 U of *Taq* DNA polymerase, 1.5 μL of 10 × buffer (50 mmol KCl, 10 mmol of Tris-HCl, pH 8.3), 2.0 mmol of MgCl_2_, 200 mmol of dNTPs, and 1.0 mmol of the forward and reverse primers. The PCR program was: denaturation at 94°C for 4 min, followed by 15 cycles of 94°C for 30 s, touchdown starting at 65°C for 45 s (decreasing 1.0°C per cycle), 72°C for 1 min, 20 cycles of 94°C for 30 s, 50°C for 45 s, 72°C for 1 min, and a final extension for 10 min at 72°C. PCR products were separated in 6% denaturing polyacrylamide gels, visualized using silver staining for polyacrylamide gels, and photographed.

Based on the integrated genetic map (Maccaferri et al., 2015a,b) and wheat genome assembly, KASP PCR markers were developed for polymorphic SNPs using a similar approach to that described in Ramirez-Gonzalez et al. (2015). KASP assays were performed following the protocol of LGC Genomics using a KASP mix containing universal FRET (fluorescence resonance energy transfer) cassettes (FAM and HEX), ROX^TM^ passive reference dye, *Taq* polymerase, free nucleotide, and MgCl_2_ in an optimized buffer. End-point fluorescence data were visualized with a microplate reader (FLUOstar Omega, BMG LABTECH, Germany) and analyzed by Klustering Caller software^7^. Reaction mixtures consisted of final volumes of 5 μL containing 2.5 μL of 2× KASP V4.0 Mastermix (LGC Genomics), 0.056 μL of assay primer mix (12 mM of each allele-specific primer and 30 mM of common primer) and 50–100 ng of genomic DNA. An S1000 Thermal Cycler (Bio-Rad) was used with cycling conditions: 94°C for 15 min, nine cycles of 94°C for 20 s, touchdown starting at 65°C for 60 s (decreasing 0.8°C per cycle), 32 cycles of 94°C for 20 s, and 57°C for 60 s ^8^.

### Genetic Map Construction and QTL Analysis

Genotypic data from our populations were used to construct a genetic map with the regression function in JoinMap version 4.0 (Van Ooijen, 2006). Recombination fractions were converted to centiMorgans (cM) using the Kosambi function (Kosambi, 1943) and a LOD score of 3.0 was used as a threshold. The linkage map was graphically visualized with Mapchart V2.3 (Voorrips, 2002). The resistance locus was assigned to a chromosomal bin by location of tightly flanking markers based on the deletion maps of Sourdille et al. (2004). QTL mapping was conducted with QTL IciMapping V4.1 (Wang, 2009) ^9^ using an inclusive composite interval mapping (ICIM) analysis. A logarithm of odds (LOD) threshold of 2.5 was set to declare a significant QTL. The MDS score for each F_2:3_ line and rAUDPC score for each F_6_ RIL at each field site were used in the respective QTL analyses. The phenotypic variances explained (*PVE*) by individual QTL were also obtained using ICIM.

### Identification of Candidate Genes and Comparative Genomics Analysis

The polymorphic SNP markers located in the target region were blasted on the Ensemble Plants website^10^ to find Gene Identifers (IDs) that were used for gene annotation from the Human-Readable Description data for gene function ^11^. Each corresponding wheat gene sequence was also used to perform a BLAST search against the genome sequence databases of *Brachypodium*^12^ and rice^13^ to identify orthologous gene sets.

## Results

### Phenotypic Evaluation

In greenhouse experiments with *Pst* races CYR31, CYR32, CYR33, V26/CH42 and V26/Gui22-9, Napo 63 was susceptible (IT 8–9) in seedling tests but highly resistant (IT 1) in adult-plant stage tests. The susceptible parent, AvS was susceptible (IT 8–9) at both growth stages. In field tests, stripe rust developed well across environments. F_1_ plants displayed IT 1-2 and DS 10–35 (necrotic flecks, without sporulation) at Yangling (**Figure 1A**) during 2014–2016 indicating that APR in Napo 63 was partially dominant. Napo 63 was rated with a mean MDS of ≤5% or a mean rAUDPC value of <5%, whereas AvS had a mean MDS ≥ 95% or a mean rAUDPC value of 100% in all environments. The frequency distributions of mean MDS for the 221 F_2:3_ lines in two environments and mean rAUDPC for 175 F_5:6_ RILs in three environments revealed continuous variation (**Figures 1B,C**), indicating quantitative inheritance. MDS data of F_2:3_ lines were significantly correlated between the two environments, with correlation coefficients of 0.90 (*P* < 0.0001) (**Table 1**), and heritability was 0.80 (**Table 2**). The rAUDPC data for the F_5:6_ RILs were highly correlated across three environments (*r* = 0.77–0.92, *P* < 0.01) (**Table 1**), and heritability was 0.87 (**Table 2**). ANOVA revealed significant differences (*P* < 0.001) in MDS and rAUDPC between lines, environments, replicates within environments and line × environment interactions (**Table 2**). The results suggested that the expression of APR was consistent across the different environments.

**Table 1:** The correlation analysis of the mean maximum disease severity (MDS) for F_2:3_ lines across two environment and relative area under the disease progress curve (rAUDPC) for F_6_ recombinant inbred line population across three environment.

Environment	Yangling 2015	Tianshui 2015	Yangling 2016	Tianshui 2016	Jiangyou 2016
Yangling 2015	1				
Tianshui 2015	0.896^∗∗∗^	1			
Yangling 2016			1		
Tianshui 2016			0.767^∗∗∗^	1	
Jiangyou 2016			0.822^∗∗∗^	0.922^∗∗∗^	1

*^∗∗∗^Significant at *P* < 0.001.*

**Table 2:** Variance components of MDS scores for F_2:3_ lines and rAUDPC for F_6_ recombinant inbred line population derived from AvS/Napo 63.

Source of variation	F_2:3_ lines		F_6_ RILs	
	Df	Mean square	Df	Mean square
Lines	221	1633.78^∗∗∗^	174	0.669^∗∗∗^
Replicates (environments)	4	265.59^∗∗∗^	6	0.117^∗∗∗^
Environments	1	2661.93^∗∗∗^	2	0.422^∗∗∗^
Lines × Environments	220	149.86^∗∗∗^	343	0.042^∗∗∗^
Error	445	63.00	1020	0.013
$\sigma_{g}^{2}$	285.82		0.055	
$h_{b}^{2}$	0.80		0.87	

*^∗∗∗^Significant at *P* < 0.001.*

### Mapping QTL for Stripe Rust Resistance with F_2:3_ Lines

A total of 509 SNPs showed polymorphisms between the DNA bulks after genotyping by the 90K SNP array; 275 of these SNPs were located on chromosome 2B and the others were distributed across other chromosomes (**Figure 1D**). Most of the SNPs on 2B were within an interval of 50–250 Mb (**Figure 1E**). Sixty four chromosome-specific SNPs in the region were selected for conversion to KASP markers and then screened on the parents and bulks to confirm polymorphisms before being genotyped on the entire population; 49 failed to distinguish the contrasting parents and bulks. Four of 76 published wheat SSR markers on chromosome 2B were also polymorphic between parents and bulks.

**FIGURE 2:**
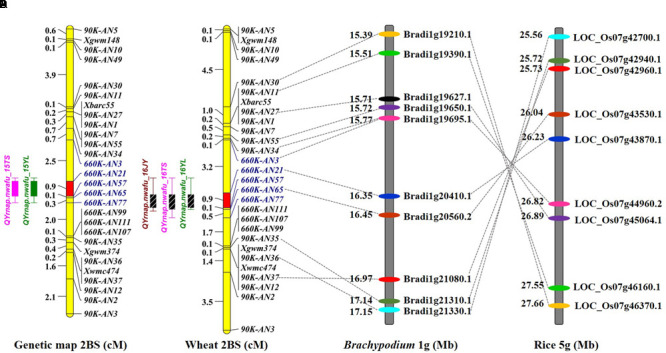
**Comparative genetic linkage maps of stripe rust resistance gene *Qyrnap.nwafu-2BS*.** The QTL region for *Qyrnap.nwafu-2BS* was identified by QTL mapping using phenotypic and marker data from AvS × Napo 63. **(A)** Genetic linkage maps of *Qyrnap.nwafu-2BS* on chromosome 2BS produced from results of by using F_2:3_ lines **(A)** and F_2:6_ RILs **(B)**. **(C,D)** Orthologous genomic regions of *Qyrnap.nwafu-2BS* on *Brachypodium* chromosome 1 and rice chromosome 5.

**FIGURE 3:**
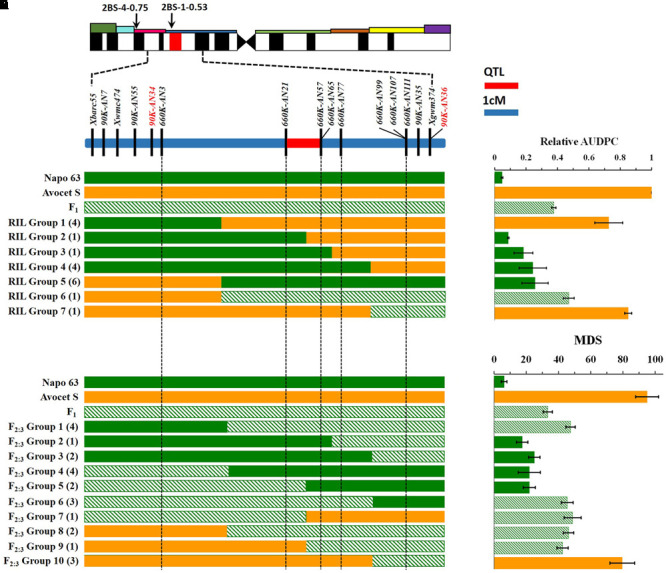
**Interval mapping of the QTL region for *Qyrnap.nwafu-2BS* and association with phenotype. (A)** Deletion bin location of *Qyrnap.nwafu-2BS* in 2BS indicated by SSR markers and linkage map of SNP and SSR markers across the *Qyrnap.nwafu-2BS* interval. **(B)** The graphical genotype of in Avocet S × Napo 63 RILs and **(D)** F_2:3_ lines are aligned against their response to stripe rust (**C,E**, respectively). Recombinants are grouped by adjacent markers, and numbers of lines in each group are indicated in parentheses. Green, orange and slash bars represent homozygous resistant (Napo 63), homozygous susceptible (Avocet S) and heterozygous resistant (F_1_) genotypes, respectively. Response groups are differentiated as susceptible (orange), moderately resistant (slash) or resistant (green) based on statistical comparison with the parental and F_1_ controls. Error bars are SEM. Detailed data are provided in Supplementary Tables S4, S5.

A genetic map was constructed using the 4 SSR and 15 KASP markers genotyped on the 224 F_2_ individuals, resulting in a linkage group spanning 16.1 cM. A major effect QTL *Qyrnap.nwafu-2BS* was placed in this map by ICIM using the mean MDS data across environments. The QTL was preliminarily located between the KASP markers *90K-AN34* (Kukri_c36026_68) and *90K-AN36* (wsnp_Ex_c62844_62315607) in an interval of 5.46 cM (**Figures 2A, 3A**).

### Validation of the QTL Using F_5:6_ RILs and Fine Mapping of *Qyrnap.nwafu-2BS*

A second genetic map was constructed for the RIL population using the same markers. (**Figures 2A,B**). The major QTL for stripe rust resistance was also detected by ICIM using mean rAUDPC data across environments (**Figure 2B**). Approximately, 7,000 SNPs showed polymorphisms in BSA with the 660K SNP array; 5,263 were located on all 21 chromosomes based on the w7984/Opata85 map (**Figure 1F**) (Jizeng Jia, personal communications). More than 130 of 2,689 SNPs in this map occurred in the chromosome 2B interval *90K-AN34*—*90K-AN36* according to the wheat genome assembly (**Figure 1G**). To fine map *Qyrnap.nwafu-2BS* and develop improved markers for routine breeding applications 14 more closely linked chromosome-specific SNP markers were converted to KASP assays and eight polymorphic KASP markers were used to map the F_2:3_ and F_5:6_ populations. All the sequences of KASP markers are listed in Supplementary Table S1.

**FIGURE 4:**
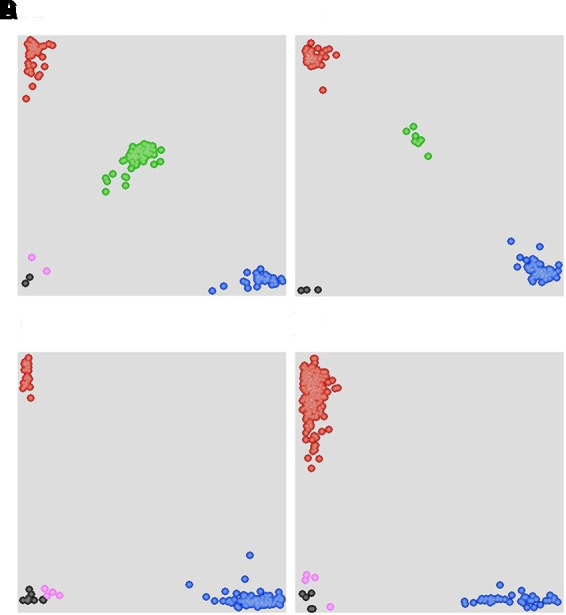
**Scatter plots for selected KASP assays showing clustering of F_2_ and F_5:6_ lines on the *X*- (FAM) and *Y*- (HEX) axes. (A)** Marker *90K-AN34* results for the F_2:3_ population showing three distinct clusters. The central cluster (green) is comprised of heterozygous individuals, whereas clusters near the axes are homozygous for either AVS (HEX; red) or Napo 63 (FAM; blue). Black dots represent the NTC (non-template control) and pink dots represent missing or failed data. **(B)** Marker *660K-AN21* results for F_5:6_ population showing two distinct clusters. **(C)** Marker *660K- AN65* results for 215 cultivars and landraces showing two distinct clusters. The clusters near the Y-axis are homozygous for the Napo 63 allele (HEX; red). **(D)** Marker *660K-AN3* for 215 cultivars and landraces showing two distinct clusters.

*Qyrnap.nwafu-2BS* was confirmed by analyzing recombinants between KASP markers *90K-AN34* and *90K-AN36*. Eighteen and 23 recombinants from F_5:6_ RILs and F_2:3_ lines were grouped based on their haplotypes across the marker interval (**Figures 3B,D**). The corresponding *Pst* phenotypes of each group were displayed using the mean rAUDPC or MDS data (**Figures 3C,E**). The mean value and standard deviation of each group were calculated on basis of the individual rAUDPC or MDS of each line (Supplementary Tables S4, S5). With the help of these more closely linked markers, this QTL region was narrowed down to a 0.9 cM interval flanked by KASP markers *660K-AN21* (AX-109302096) and *660K-AN57* (AX-110457023) (**Figures 2A,B, 3A**). This explained 62.9 and 69.2% of the phenotypic variances in average MDS among F_2:3_ lines grown in two environments. For the F_5:6_ RILs the corresponding variances in rAUDPC explained ranged from 45.7 to 62.4% in three environments (**Table 3**). According to the position of the SSR markers in the 2B deletion bin map, the QTL was located in the 2BS-1-0.53 region (**Figure 3A**). **Figures 4A,B** showed genotypic data of F_2:3_ and F_5:6_ lines from the KASP assays.

**Table 3:** Summary of stripe rust resistance QTL detected by ICIM in the AvS × Napo 63 F_2:3_ population across two environments and F_5:6_ population across three environments.

Location and year	Marker interval	LOD^a^	Add^b^	PVE^c^
**MDS**				
Yangling 2015	*660K-AN21—660K-AN57*	48.6	–27.3	62.9
Tianshui 2015	*660K-AN21—660K-AN57*	56.1	–26.1	69.2
Average				66.1
**rAUDPC**				
Yangling 2016	*660K-AN21—660K-AN57*	22.7	–0.18	45.7
Tianshui 2016	*660K-AN21—660K-AN57*	36.0	–0.26	62.4
Jiangyou 2016	*660K-AN21—660K-AN57*	34.6	–0.24	59.1
Average				55.7

*^a^LOD, logarithm of odds score.*

*^b^Add, additive effect of resistance allele.*

*^c^PVE, percentages of the phenotypic variance explained by individual QTL.*

### Evaluation of Linked Markers in Various Wheat Genotypes

A set of 215 wheat genotypes was used to evaluate the robustness of KASP markers derived from *660K-AN3, 660K-AN21, 660K-AN57*, and *660K-AN65* KASP in predicting the presence of *Qyrnap.nwafu-2BS* in the mapping population. The *660K-AN57* KASP marker did not detect the presence/absence of the resistance allele accurately and resulted in a high level of misclassification (Supplementary Table S2). Markers *660K-AN3, 660K-AN21*, and *660K-AN65* amplified 1, 2, and 12 false positives among susceptible cultivars, respectively (**Figures 4C,D**), but no genotype had all SNP markers (**Table 4**), indicating that they should not be used alone to detect *Qyrnap.nwafu-2BS*. Ten cultivars with APR contained the same *660K-AN3, 660K-AN21*, and *660K-AN65* alleles, as Napo 63. They were CIMMYT lines Buc/Bjy, Kariega, Kenya Kudu, Louise, Bluejay“S”, Luke, Mos“S”-Imu, Opata 85, Taa 72 and Taa 73 indicating that they may have the resistance gene. Nonetheless, further genetic studies need to be performed to confirm allelism or otherwise. APR gene or QTL on chromosome 2BS have been identified in Kariega, Kenya Kudu, Louise, Luke and Opata 85.

**Table 4:** Phenotype and alleles of KASP markers flanking *Qyrnap.nwafu-2BS* in Napo 63, Avocet S (AvS), susceptible checks and 19 wheat cultivars and landraces.

Wheat line	KASP markers				Severity (%) and reaction at adult-plant stage^b^					
	*660K-AN3*	*660K-AN21*	*660K-AN57*	*660K-AN65*	Yangling			Tianshui		
					2014	2015	2016	2014	2015	2016
Napo 63	^a^AA	GG	TT	TT	5R	1R	5R	5R	5R	5R
Avocet S	CC	AA	CC	CC	100S	100S	100S	100S	100S	100S
XY22 (CK)	CC	AA	CC	CC	100S	95S	100S	100S	100S	100S
MX169 (CK)	CC	AA	CC	CC	100S	100S	100S	100S	100S	100S
IDO444	CC	AA	TT	CC	–	–	–	–	–	–
Kariega	AA	GG	TT	TT	–	–	–	–	–	–
Kenya Kudu	AA	GG	TT	TT	–	–	–	–	–	–
Luke	AA	GG	TT	TT	–	–	–	–	–	–
Louise	AA	GG	TT	TT	–	–	–	–	–	–
Opata 85	AA	GG	TT	TT	–	–	–	–	–	–
Buc/Bjy	AA	GG	TT	TT	1R	1R	5R	5R	10R	5R
Bluejay“S”	AA	GG	TT	TT	1R	1R	1R	1R	5R	10R
Mos“S”-Imu	AA	GG	TT	TT	1R	1R	5R	10R	5R	5R
Taa 72	AA	GG	TT	TT	10R	10R	5R	15R	10R	5R
Taa 73	AA	GG	TT	TT	15MR	10R	10R	10R	5R	5R
Yang 11–59	CC	GG	TT	TT	70MS	90S	100S	90S	80MS	100S
Zhouyuan 9369	AA	GG	TT	CC	40MR	50MS	40MR	30MR	40MS	50MR
Jimai 41	CC	AA	TT	TT	80S	90S	80S	100S	90S	90S
Jimai 44	CC	AA	TT	TT	70S	90S	80S	90S	100S	100S
Jimai 45	CC	AA	TT	TT	80S	100S	90S	100S	90S	100S
Luo 8112	CC	AA	TT	TT	70S	90S	80S	100S	100S	100S
Shengnong 1	CC	AA	TT	TT	60MS	80MS	90S	70MS	80S	80S
Suzhou 1310	CC	AA	TT	TT	70MS	90MS	70MS	80S	90S	100S
Suzhou 1313	CC	AA	TT	TT	60MS	80S	80S	90S	100S	90S
Suzhou 22	CC	AA	TT	TT	80MS	90S	100S	80S	100S	100S
Wanximai 2013–20	CC	AA	CC	TT	70S	100S	90S	90S	90S	100S
Wanximai 2013–9	CC	AA	CC	TT	80S	100S	70S	80S	90S	90S
Zhongmai 170	CC	AA	TT	TT	80S	90MS	80S	90S	70S	80S

*^a^AA means the SNP locus.*

*^b^A field response was a combination of disease severity recorded as a single value for each accession at each site from 0 to 100% of the foliage infected using Peterson et al. (1948) scale, and R, resistant; MR, moderately resistant; M, moderately resistant to moderately susceptible; MS, moderately susceptible; and S, susceptible.*

### Comparative Genomic Analysis and Candidate Genes in the Target Genomic Region

To assess collinearity of the target genomic region in wheat with *Brachypodium* and rice, the relevant wheat gene sequences were selected to identify orthologous genes in comparative genomic regions in *Brachypodium* and rice. For simplification only 18 polymorphic KASP-SNP markers located between *90K-AN30* and *90K-AN12* are listed (**Figures 2C,D**). Comparative analyses revealed that 11 KASP markers in wheat showed collinearity with *Brachypodium* and rice. Ten predicted *Brachypodium* genes, except *Bradi1g19627.1*, in this region have orthologous genes in rice, but arranged in a reverse order indicating an inversion in rice compared to *Brachypodium* and wheat. The target region spanned a 4.3 Mb interval from 154.9 to 159.2 Mb in the wheat genome assembly, which has synteny with a 102.2 kb genomic region *Brachypodium* chromosome 1S (from *Bradi1g20410.1* to *Bradi1g20560.2*) and a 188.4 kb region in rice chromosome 5S (from *LOC_Os07g43530.1* to *LOC_Os07g43870.1*). A total of 67 SNP markers were blasted in the target region of wheat and 14 wheat genes were identified; several SNPs were mapped in the same gene (Supplementary Table S3). The target region contained a glycosyltransferase gene defined by marker AX-110644789 (gene *Traes_2BS_2B483208E*) that is possibly involved in plant disease resistance (Bolouri Moghaddam and Van den Ende, 2012) and therefore can be regarded as a potential candidate gene for *Qyrnap.nwafu-2BS*.

## Discussion

The resistance gene-rich chromosome 2BS region is known to possess many genes for both ASR and APR, including *Yr27, Yr31, Yr41, YrC51, YrF, YrH9014, YrKK, YrP81, YrSp*, and *YrTp1* (Maccaferri et al., 2015b). Resistances conferred by *Yr27, Yr31, Yr41, YrP81*, and *YrSp* have been overcome by Chinese *Pst* races (Zheng et al., 2014; Zeng et al., 2015). All confer all-stage or race-specific resistance. However, *YrKK*, characterized in the CIMMYT-derived common wheat cultivar Kenya Kudu (Kenya 131/Kenya 184P), confers immunity in adult-plants and had a small effect on seedling response (Li et al., 2013). Pedigree analyses revealed that Napo 63 has a distant relationship with Kenya Kudu through the common parent Florence (**Supplementary Figure S1**). Several stripe rust QTLs with major APR effects for on chromosome 2BS have been reported (Rosewarne et al., 2013). For example, *QYrlu.cau-2BS1* and *QYrlu.cau-2BS2* in Luke, *QYrid.ui-2B.1* and *QYrid.ui-2B.2* in IDO444, *QYr.sgi-2B.1* in Kariega, *QYrlo.wpg-2BS* in Louise, *QYr-2B* in Opata 85 and *QYr.inra-2B.1* in Camp Remy conferring large effects were located in a similar region (Boukhatem et al., 2002; Mallard et al., 2005; Guo et al., 2008; Carter et al., 2009; Prins et al., 2011; Chen et al., 2012). Based on the integrative genetic map (Maccaferri et al., 2015b) all of the above major QTLs were within the interval 19.9–50.2 cM, within which we placed *Qyrnap.nwafu-2BS* between 40.02 and 43.44 cM. Pedigree analyses indicated that all cultivars except IDO444 were derived from CIMMYT germplasm^14^. IDO444 (PI 578278) is a winter wheat developed by the University of Idaho wheat breeding program in 1994 without CIMMYT germplasm in its pedigree (XM Chen, personal communication ^15^). Moreover, in our study, the same alleles of KASP markers *660K-AN3, 660K-AN21, 660K-AN57*, and *660K-AN65* were detected in Kariega, Kenya Kudu, Louise, Luke and Opata 85. Based on molecular detection assays, origin, and chromosome location it appears likely that the same gene is present in at least some of these varieties.

Although BSA has a wide range of applications in genetics and genomics, it can also be used for dissection of relatively simple traits controlled by major genes (Sun et al., 2010; Zou et al., 2016). For quantitative traits that are controlled by polygenes with different effects, BSA can be improved through increasing population size and marker density, using multiple bulked samples, and precision phenotyping. In this study, F_2:3_ lines and F_5:6_ RILs were chosen for genotyping by 90 and 660K SNP assays using segregates with extreme differences in phenotype across all environments (**Figure 1**). A large number of SNPs associated with the resistance were then selected. Based on SNP location a major QTL region was identified and chromosome-specific SNPs were selected and converted to KASP markers for further screening by BSA. Fifteen KASP markers developed from 64 SNPs from the 90K SNP assay were employed to generate a genetic map and the resistance locus was located in an interval of 5.46 cM spanned by *90K-AN34* and *90K-AN36*. The same procedure was used to fine map the QTL with the 660K SNP array and *Qyrnap.nwafu-2BS* was narrowed to a 0.9 cM interval between SNP markers *660K-AN21* and *660K-AN57*. Thus, validation of SNP arrays with KASP-SNP assays improves the accuracy of fine mapping in QTL regions. Comparative genomics showed that the target genomic region has synteny with *Brachypodium* and rice, and except that the region was inverted in rice, there were no rearrangements to complicate future map-based cloning.

To date, three adult-plant stripe rust resistance genes have been cloned. *Yr18* encodes an ATP-binding cassette (ABC) transporter (Krattinger et al., 2009), *Yr36* encodes a protein containing both kinase and START domains (Fu et al., 2009), and *Yr46* encodes a hexose transporter that inhibits hexose uptake from the apoplast by host cells (Moore et al., 2015). Based on the functions of the identified proteins, vesicle trafficking and protein/metabolite transportation are probably common physiological processes involved in APR (Niks et al., 2015). In this study, bioinformatics analysis of the mapped SNPs in the target region for stripe rust resistance showed that wheat gene *Traes_2BS_2B483208E* encoded a glycosyltransferase. Sugars are involved in many metabolic and signaling pathways in plants. Sugar signaling may also contribute to immune responses against pathogens when changing concentrations or ratios of sugars in plant tissue can induce plant defense genes, influence plant hormone pathways, and induce resistance to various diseases (Bolouri Moghaddam and Van den Ende, 2012; Dodds and Lagudah, 2016). *Traes_2BS_2B483208E* function in energy metabolism and transport is similar to that of *Lr67/Yr46.* Prediction of candidate genes sets a basis for the next step in map-based cloning. Nevertheless, further genetic studies and more detailed analyses are needed to confirm the roles of this and other candidate genes in stripe rust response.

Marker-assisted selection provides an efficient way to incorporate and pyramid genes in breeding programs. However, the markers must be reliable, specific, and easily used in an economic way. This is particularly true for disease resistance where remotely located disease nurseries may be needed for phenotyping to ensure reliable and repeatable results (Chen, 2013). KASP genotyping technology provides a high throughput platform at low cost. In this study, we identified *660K-AN3, 660K-AN21*, and *660K-AN65* as flanking markers suitable for selection of *Qyrnap.nwafu-2BS* but when tested on a set of diverse genotypes the markers were insufficiently robust to determine presence or absence of *Qyrnap.nwafu-2BS* and therefore should be used together.

## Author Contributions

JW: conducted the experiments, analyzed the data, and wrote the manuscript. QW and DH: identified the resistant parental line, made the cross and participated in the field experiments. SL, SH, and JM: participated in field experiments and contributed to the genotyping experiment. QW and QZ: assisted in analyzing the data. LH: revised the manuscript. DH and ZK: conceived and directed the project and revised the manuscript.

## Conflict of Interest Statement

The authors declare that the research was conducted in the absence of any commercial or financial relationships that could be construed as a potential conflict of interest.

## Footnotes

1
http://www.Lgcgenomics.com

2
http://wgb.cimmyt.org/gringlobal/search.aspx

3
http://www.isbreeding.net/software/default.aspx?type=detail&type2=detail2&id=18

4
http://www.capitalbio.com

5
http://www.illumina.com

6
http://www.wheatgenome.org/

7
http://www.lgcgroup.com/

8
http://www.cerealsdb.uk.net/cerealgenomics/CerealsDB/PDFs/KASP_SNP_Genotyping_Manual.pdf

9
http://www.isbreeding.net/software/default.aspx?type=detail&type2=detail2&id=18

10
http://plants.ensembl.org/index.html

11
http://plants.ensembl.org/Triticum_aestivum/Info/Index

12
http://pgsb.helmholtz-muenchen.de/plant/brachypodium/index.jsp

13
http://rice.plantbiology.msu.edu/

14
http://wgb.cimmyt.org/gringlobal/search.aspx

15
https://npgsweb.ars-grin.gov/gringlobal/accessiondetail.aspx?id=1473249

## Supplementary Material

FIGURE S1 **Pedigrees of Napo 63 and Kenya Kudu**.

## References

[B1] Abe A. Kosugi S. Yoshida K. Natsume S. Takagi H. Kanzaki H. (2012). Genome sequencing reveals agronomically important loci in rice using MutMap. *Nat. Biotechnol.* 30 174–178. 10.1038/nbt.2095 22267009

[B2] Allard R. W. (1960). *Princilpes of Plant Breeding.* New York, NY: John Wiley and Sons.

[B3] Allen A. M. Barker G. L. A. Wilkinson P. Burridge A. Winfield M. Coghill J. (2013). Discovery and development of exome-based, co-dominant single nucleotide polymorphism markers in hexaploid wheat (*Triticum aestivum* L.). *Plant Biotechnol. J.* 11 279–295. 10.1111/pbi.12009 23279710

[B4] Allen A. M. Winfield M. O. Burridge A. J. Downie R. C. Benbow H. R. Barker G. L. A. (2016). Characterization of a Wheat Breeders’ Array suitable for high-throughput SNP genotyping of global accessions of hexaploid bread wheat (*Triticum aestivum*). *Plant Biotechnol. J.* 15 390–401. 10.1111/pbi.12635 27627182 PMC5316916

[B5] Barabaschi D. Tondelli A. Desiderio F. Volante A. Vaccino P. Valè G. (2016). Next generation breeding. *Plant Sci.* 242 3–13. 10.1016/j.plantsci.2015.07.010 26566820

[B6] Bolouri Moghaddam M. R. Van den Ende W. (2012). Sugars and plant innate immunity. *J. Exp. Bot.* 63 3989–3998. 10.1093/jxb/ers129 22553288

[B7] Boukhatem N. Baret P. V. Mingeot D. Jacquemin J. M. (2002). Quantitative trait loci for resistance against Yellow rust in two wheat-derived recombinant inbred line populations. *Theor. Appl. Genet.* 104 111–118. 10.1007/s001220200013 12579435

[B8] Brown J. K. (2015). Durable resistance of crops to disease: a Darwinian perspective. *Annu. Rev. Phytopathol.* 53 513–539. 10.1146/annurev-phyto-102313-045914 26077539

[B9] Carter A. H. Chen X. M. Garland-Campbell K. Kidwell K. K. (2009). Identifying QTL for high-temperature adult-plant resistance to stripe rust (*Puccinia striiformis* f. *sp. tritici*) in the spring wheat (*Triticum aestivum* L.) cultivar ‘Louise’. *Theor. Appl. Genet.* 119 1119–1128. 10.1007/s00122-009-1114-2 19644666

[B10] Cavanagh C. R. Chao S. M. Wang S. C. Huang B. E. Stephen S. Kiani S. (2013). Genome-wide comparative diversity uncovers multiple targets of selection for improvement in hexaploid wheat landraces and cultivars. *Proc. Natl. Acad. Sci. U.S.A.* 110 8057–8062. 10.1073/pnas.1217133110 23630259 PMC3657823

[B11] Chen J. Chu C. Souza E. J. Guttieri M. J. Chen X. M. Xu S. (2012). Genome-wide identification of QTL conferring high-temperature adult-plant (HTAP) resistance to stripe rust (*Puccinia striiformis* f. sp *tritici*) in wheat. *Mol. Breed.* 29 791–800. 10.1007/s11032-011-9590-x

[B12] Chen W. Q. Wu L. R. Liu T. G. Xu S. C. Jin S. L. Peng Y. L. (2009). Race dynamics, diversity, and virulence evolution in *Puccinia striiformis* f. sp. *tritici*, the causal agent of wheat stripe rust in China from 2003 to 2007. *Plant Dis.* 93 1093–1101. 10.1094/Pdis-93-11-1093 30754577

[B13] Chen X. M. (2005). Epidemiology and control of stripe rust [*Puccinia striiformis* f. sp. *tritici*] on wheat. *Can. J. Plant Pathol.* 27 314–337. 10.1080/07060660509507230

[B14] Chen X. M. (2013). Review article: high-temperature adult-plant resistance, key for sustainable control of stripe rust. *Am. J. Plant Sci.* 04 608–627. 10.4236/ajps.2013.43080

[B15] Chen X. M. Line R. F. (1995). Gene number and heritability of wheat cultivars with durable, high-temperature, adult-plant (HTAP) resistance and interaction of HTAP and race-specific seedling resistance to *Puccinia striiformis*. *Phytopathology* 85 573–578. 10.1094/Phyto-85-567

[B16] Chen X. M. Penman L. Wan A. M. Cheng P. (2010). Virulence races of *Puccinia striiformis* f. sp *tritici* in 2006 and 2007 and development of wheat stripe rust and distributions, dynamics, and evolutionary relationships of races from 2000 to 2007 in the United States. *Can. J. Plant Pathol.* 32 315–333. 10.1080/07060661.2010.499271

[B17] Dodds P. N. Lagudah E. S. (2016). Starving the enemy. *Science* 354 1377–1378. 10.1126/science.aak9460 27980171

[B18] Ellis J. G. Lagudah E. S. Spielmeyer W. Dodds P. N. (2014). The past, present and future of breeding rust resistant wheat. *Front. Plant Sci.* 5:641. 10.3389/fpls.2014.00641 PMC4241819 25505474

[B19] Forrest K. Pujol V. Bulli P. Pumphrey M. Wellings C. Herrera-Foessel S. (2014). Development of a SNP marker assay for the *Lr67* gene of wheat using a genotyping by sequencing approach. *Mol. Breed.* 34 2109–2118. 10.1007/s11032-014-0166-4

[B20] Fu D. L. Uauy C. Distelfeld A. Blechl A. Epstein L. Chen X. M. (2009). A kinase-START gene confers temperature-dependent resistance to wheat stripe rust. *Science* 323 1357–1360. 10.1126/science.1166289 19228999 PMC4737487

[B21] Guo Q. Zhang Z. J. Xu Y. B. Li G. H. Feng J. Zhou Y. (2008). Quantitative trait loci for high-temperature adult-plant and slow-rusting resistance to *Puccinia striiformis* f. sp *tritici* in wheat cultivars. *Phytopathology* 98 803–809. 10.1094/PHYTO-98-7-0803 18943256

[B22] Han D. J. Zhang P. Y. Wang Q. L. Zeng Q. D. Wu J. H. Zhou X. L. (2012). Identification and evaluation of resistance to stripe rust in 1980 wheat landraces and abroad germplasm. *Sci. Agric. Sin.* 45 5013–5023. 10.3864/j.issn.0578-1752.2012.24.006

[B23] Hou L. Chen X. M. Wang M. N. See D. R. Chao S. M. Bulli P. (2015). Mapping a large number of QTL for durable resistance to stripe rust in winter wheat Druchamp using SSR and SNP markers. *PLoS ONE* 10:e0126794. 10.1371/journal.pone.0126794 PMC4430513 25970329

[B24] Hovmøller M. S. Walter S. Justesen A. F. (2010). Escalating threat of wheat rusts. *Science* 329 369–369. 10.1126/science.1194925 20651122

[B25] Jia J. Z. Zhao G. Y. (2016). *Wheat660 SNP Array Developed by CAAS.* Available at: http://wheat.pw.usda.gov/ggpages/topics/Wheat660_SNP_array_developed_by_CAAS.pdf

[B26] Kang Z. S. Zhao J. Han D. J. Zhang H. C. Wang X. J. Wang C. F. (2010). “Status of wheat rust research and control in China,” in *Proceedings of the BGRI 2010 Technical Workshop Oral Presentations* Saint Petersburg 50–69.

[B27] Kosambi D. D. (1943). The estimation of map distances from recombination values. *Ann. Hum. Genet.* 12 172–175. 10.1111/j.1469-1809.1943.tb02321.x

[B28] Krattinger S. G. Jordan D. R. Mace E. S. Raghavan C. Luo M. C. Keller B. (2013). Recent emergence of the wheat *Lr34* multi-pathogen resistance: insights from haplotype analysis in wheat, rice, sorghum and *Aegilops tauschii*. *Theor. Appl. Genet.* 126 663–672. 10.1007/s00122-012-2009-1 23117720

[B29] Krattinger S. G. Lagudah E. S. Spielmeyer W. Singh R. P. Huerta-Espino J. McFadden H. (2009). A putative ABC transporter confers durable resistance to multiple fungal pathogens in wheat. *Science* 323 1360–1363. 10.1126/science.1166453 19229000

[B30] Li Z. F. Singh S. Singh R. P. López-Vera E. E. Huerta-Espino J. (2013). Genetics of resistance to yellow rust in PBW343 × Kenya Kudu recombinant inbred line population and mapping of a new resistance gene *YrKK*. *Mol. Breed.* 32 821–829. 10.1007/s11032-013-9909-x

[B31] Li Z. Q. Zeng S. M. (2002). *Wheat Rust in China.* Beijing: China Agriculture Press.

[B32] Lin F. Chen X. M. (2007). Genetics and molecular mapping of genes for race-specific all-stage resistance and non-race-specific high-temperature adult-plant resistance to stripe rust in spring wheat cultivar Alpowa. *Theor. Appl. Genet.* 114 1277–1287. 10.1007/s00122-007-0518-0 17318493

[B33] Line R. F. Qayoum A. (1992). Virulence, aggressiveness, evolution, and distribution of races of *Puccinia striiformis* (the cause of stripe rust of wheat) in North America 1968-1987. Washington, DC: US Department of Agriculture 74.

[B34] Liu J. D. He Z. H. Wu L. Bai B. Wen W. E. Xie C. J. (2015). Genome-wide linkage mapping of QTL for adult-plant resistance to stripe rust in a Chinese wheat population Linmai 2 x Zhong 892. *PLoS ONE* 10:e0145462. 10.1371/journal.pone.0145462 PMC4694644 26714310

[B35] Liu W. Z. Maccaferri M. Bulli P. Rynearson S. Tuberosa R. Chen X. M. (2016). Genome-wide association mapping for seedling and field resistance to *Puccinia striiformis* f. sp. *tritici* in elite durum wheat. *Theor. Appl. Genet.* 130 649–667. 10.1007/s00122-016-2841-9 28039515

[B36] Maccaferri M. Ricci A. Salvi S. Milner S. G. Noli E. Martelli P. L. (2015a). A high-density, SNP-based consensus map of tetraploid wheat as a bridge to integrate durum and bread wheat genomics and breeding. *Plant Biotechnol. J.* 13 648–663. 10.1111/pbi.12288 25424506

[B37] Maccaferri M. Zhang J. L. Bulli P. Abate Z. Chao S. M. Cantu D. (2015b). A genome-wide association study of resistance to stripe rust (*Puccinia striiformis* f. sp. *tritici*) in a worldwide collection of hexaploid spring wheat (*Triticum aestivum* L.). *G*3. 5 449–465. 10.1534/g3.114.014563 25609748 PMC4349098

[B38] Mallard S. Gaudet D. Aldeia A. Abelard C. Besnard A. L. Sourdille P. (2005). Genetic analysis of durable resistance to yellow rust in bread wheat. *Theor. Appl. Genet.* 110 1401–1409. 10.1007/s00122-005-1954-3 15841362

[B39] McIntosh R. A. Wellings C. R. Park R. F. (1995). *Wheat Rusts, an Atlas of Resistance Genes.* East Melbourne, VIC: CSIRO Publications 20–26.

[B40] Michelmore R. W. Paran I. Kesseli R. (1991). Identification of markers linked to disease-resistance genes by bulked segregant analysis: a rapid method to detect markers in specific genomic regions by using segregating populations. *Proc. Natl. Acad. Sci. U.S.A.* 88 9828–9832. 10.1073/pnas.88.21.9828 1682921 PMC52814

[B41] Moore J. W. Herrera-Foessel S. Lan C. X. Schnippenkoetter W. Ayliffe M. Huerta-Espino J. (2015). A recently evolved hexose transporter variant confers resistance to multiple pathogens in wheat. *Nat. Genet.* 47 1494–1498. 10.1038/ng.3439 26551671

[B42] Niks R. E. Qi X. Q. Marcel T. C. (2015). Quantitative resistance to biotrophic filamentous plant pathogens: concepts, misconceptions, and mechanisms. *Annu. Rev. Phytopathol.* 53 445–470. 10.1146/annurev-phyto-080614-115928 26047563

[B43] Peterson R. F. Campbell A. B. Hannah A. E. (1948). A diagrammatic scale for estimating rust intensity of leaves and stem of cereals. *Can. J. Res. Sect. C* 26 496–500. 10.1139/cjr48c-033

[B44] Prins R. Pretorius Z. A. Bender C. M. Lehmensiek A. (2011). QTL mapping of stripe, leaf and stem rust resistance genes in a Kariega × Avocet S doubled haploid wheat population. *Mol. Breed.* 27 259–270. 10.1007/s11032-010-9428-y

[B45] Ramirez-Gonzalez R. H. Uauy C. Caccamo M. (2015). PolyMarker: a fast polyploid primer design pipeline. *Bioinformatics* 31 2038–2039. 10.1093/bioinformatics/btv069 25649618 PMC4765872

[B46] Rasheed A. Wen W. E. Gao F. M. Zhai S. N. Jin H. Liu J. D. (2016). Development and validation of KASP assays for genes underpinning key economic traits in bread wheat. *Theor. Appl. Genet.* 129 1843–1860. 10.1007/s00122-016-2743-x 27306516

[B47] Rosewarne G. M. Herrera-Foessel S. A. Singh R. P. Huerta-Espino J. Lan C. X. He Z. H. (2013). Quantitative trait loci of stripe rust resistance in wheat. *Theor. Appl. Genet.* 126 2427–2449. 10.1007/s00122-013-2159-9 23955314 PMC3782644

[B48] Somers D. J. Isaac P. Edwards K. (2004). A high-density microsatellite consensus map for bread wheat (*Triticum aestivum* L.). *Theor. Appl. Genet.* 109 1105–1114. 10.1007/s00122-004-1740-7 15490101

[B49] Song W. N. Ko L. Henry R. J. (1994). Polymorphisms in the *α-amy1* gene of wild and cultivated barley revealed by the polymerase chain reaction. *Theor. Appl. Genet.* 89 509–513. 10.1007/BF00225388 24177902

[B50] Sourdille P. Singh S. Cadalen T. Brown-Guedira G. L. Gay G. Qi L. L. (2004). Microsatellite-based deletion bin system for the establishment of genetic-physical map relationships in wheat (*Triticum aestivum* L.). *Funct. Integr. Genomics* 4 12–25. 10.1007/s10142-004-0106-1 15004738

[B51] St Clair D. A. (2010). Quantitative disease resistance and quantitative resistance loci in breeding. *Annu. Rev. Phytopathol.* 48 247–268. 10.1146/annurev-phyto-080508-081904 19400646

[B52] Sun Y. Wang J. Crouch J. H. Xu Y. (2010). Efficiency of selective genotyping for genetic analysis of complex traits and potential applications in crop improvement. *Mol. Breed.* 26 493–511. 10.1007/s11032-010-9390-8

[B53] Takagi H. Abe A. Yoshida K. Kosugi S. Natsume S. Mitsuoka C. (2013). QTL-seq: rapid mapping of quantitative trait loci in rice by whole genome resequencing of DNA from two bulked populations. *Plant J.* 74 174–183. 10.1111/tpj.12105 23289725

[B54] Van Ooijen J. W. (2006). *JoinMap4 Software for the Calculation of Genetic Linkage Maps in Experimental Populations.* Wageningen: Kyazma BV.

[B55] Voorrips R. E. (2002). MapChart: software for the graphical presentation of linkage maps and QTLs. *J Hered.* 93 77–78. 10.1093/jhered/93.1.77 12011185

[B56] Wan A. M. Chen X. M. He Z. H. (2007). Wheat stripe rust in China. *Crop Pasture Sci.* 58 605–619. 10.1071/AR06142

[B57] Wang J. K. (2009). Inclusive composite interval mapping of quantitative trait genes. *Acta Agron. Sin.* 35 239–245. 10.3724/SP.J.1006.2009.00239

[B58] Wang M. Wang S. Xia G. M. (2015). From genome to gene: a new epoch for wheat research? *Trends Plant Sci.* 20 380–387. 10.1016/j.tplants.2015.03.010 25887708

[B59] Wang S. C. Wong D. Forrest K. Allen A. Chao S. M. Huang B. E. (2014). Characterization of polyploid wheat genomic diversity using a high-density 90 000 single nucleotide polymorphism array. *Plant Biotechnol. J.* 12 787–796. 10.1111/pbi.12183 24646323 PMC4265271

[B60] Wellings C. R. (2011). Global status of stripe rust: a review of historical and current threats. *Euphytica* 179 129–141. 10.1007/s10681-011-0360-y

[B61] Wiesnerhanks T. Nelson R. (2016). Multiple disease resistance in plants. *Annu. Rev. Phytopathol.* 54 8.1–8.24. 10.1146/annurev-phyto-080615-100037 27296142

[B62] Win K. T. Vegas J. Zhang C. Song K. Lee S. (2016). QTL mapping for downy mildew resistance in cucumber via bulked segregant analysis using next-generation sequencing and conventional methods. *Theor. Appl. Genet.* 130 199–211. 10.1007/s00122-016-2806-z 27714417

[B63] Winfield M. O. Allen A. M. Burridge A. J. Barker G. L. A. Benbow H. R. Wilkinson P. A. (2016). High-density SNP genotyping array for hexaploid wheat and its secondary and tertiary gene pool. *Plant Biotechnol. J.* 14 1195–1206. 10.1111/pbi.12485 26466852 PMC4950041

[B64] Wu J. H. Wang Q. L. Chen X. M. Wang M. J. Mu J. M. Lv X. N. (2016). Stripe rust resistance in wheat breeding lines developed for central Shaanxi, an overwintering region for *Puccinia striiformis* f. sp. *tritici* in China. *Can. J. Plant Pathol.* 38 317–324. 10.1080/07060661.2016.1206039

[B65] Yang H. Li C. Lam H. M. Clements J. Yan G. Zhao S. C. (2015). Sequencing consolidates molecular markers with plant breeding practice. *Theor. Appl. Genet.* 128 779–795. 10.1007/s00122-015-2499-8 25821196

[B66] Zegeye H. Rasheed A. Makdis F. Badebo A. Ogbonnaya F. C. (2014). Genome-wide association mapping for seedling and adult plant resistance to stripe rust in synthetic hexaploid wheat. *PLoS ONE* 9:e105593. 10.1371/journal.pone.0105593 PMC4143293 25153126

[B67] Zeng Q. D. Han D. J. Wang Q. L. Yuan F. P. Wu J. H. Zhang L. (2014). Stripe rust resistance and genes in Chinese wheat cultivars and breeding lines. *Euphytica* 196 271–284. 10.1007/s10681-013-1030-z

[B68] Zeng Q. D. Shen C. Yuan F. P. Wang Q. L. Wu J. H. Xue W. B. (2015). The resistance evaluation of the *Yr* genes to the main prevalent pathotypes of *Puccinia striiformis* f. sp. *tritici* in China. *Acta Phytopathol. Sin.* 45 641–650. 10.13926/j.cnki.apps.2015.06.011

[B69] Zhao J. Wang M. Chen X. Kang Z. (2016). Role of alternate hosts in epidemiology and pathogen variation of cereal rusts. *Annu. Rev. Phytopathol.* 54 9.1–9.22. 10.1146/annurev-phyto-080615-095851 27296143

[B70] Zheng J. Yan Z. Zhao L. Li S. Zhang Z. Garry R. (2014). Molecular mapping of a stripe rust resistance gene in wheat line C51. *J. Genet.* 93 443–450. 10.1007/s12041-014-0401-0 25189239

[B71] Zou C. Wang P. Xu Y. (2016). Bulked sample analysis in genetics, genomics and crop improvement. *Plant Biotechnol. J.* 14 1941–1955. 10.1111/pbi.12559 26990124 PMC5043468

