# Corrigendum: Saturation mapping of a major effect QTL for stripe rust resistance on wheat chromosome 2B in cultivar Napo 63 using SNP genotyping arrays

**DOI:** 10.3389/fpls.2025.1568966

**Published:** 2025-02-21

**Authors:** Jianhui Wu, Qilin Wang, Shengjie Liu, Shuo Huang, Jingmei Mu, Qingdong Zeng, Lili Huang, Dejun Han, Zhensheng Kang

**Affiliations:** ^1^ State Key Laboratory of Crop Stress Biology for Arid Areas, College of Plant Protection, Northwest A&F University, Yangling, China; ^2^ State Key Laboratory of Crop Stress Biology for Arid Areas, College of Agronomy, Northwest A&F University, Yangling, China

**Keywords:** adult-plant resistance, bulked segregant analysis, molecular markers, *Puccinia striiformis*, *Triticum aestivum*

In the published article, there was an error in **Figure 1** as published. The photos of Avocet S and F_1_ were not the original wheat lines. We carefully checked them and found that they are the cultivar Zhengmai 9023 and the offspring of Friedrichswerther, respectively. We got the wrong order number of the materials. The corrected **Figure 1** and its caption appear below.

**Figure 1:**
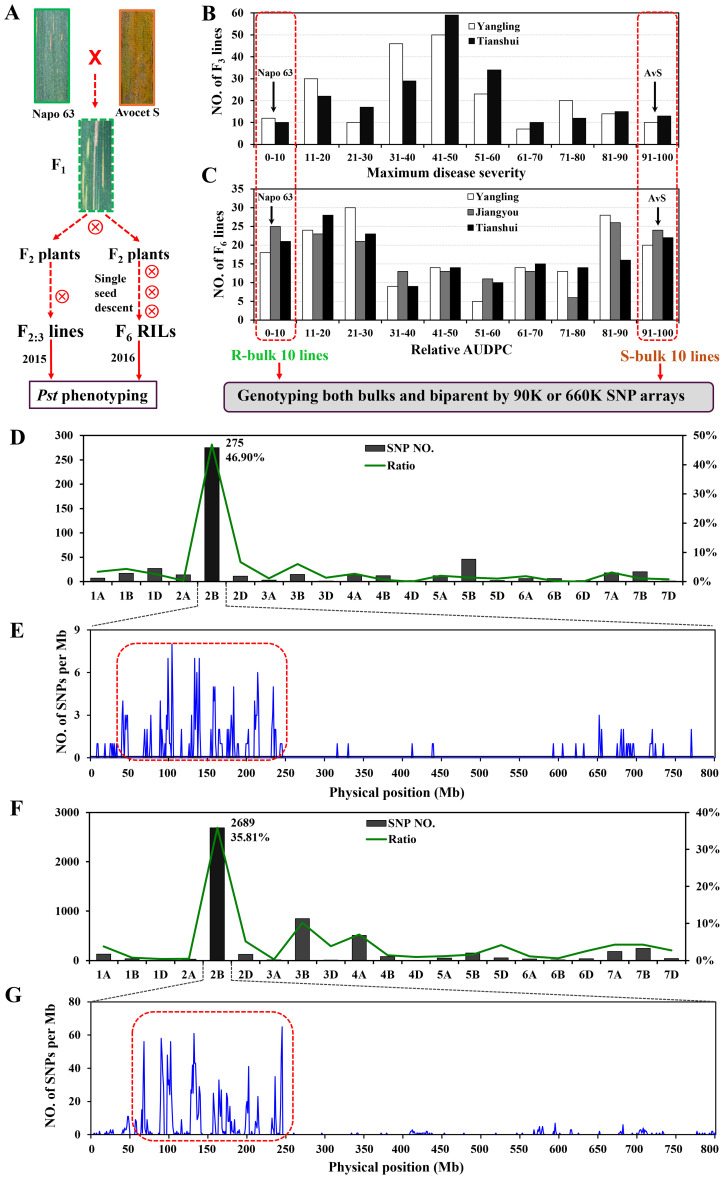
Overview of analyses. F_2:3_ lines and F_5:6_ recombinant inbred lines (RILs) were derived from the cross AvS × Napo 63. **(A)** Phenotypes of AvS, Napo 63 and their progenies across all environments and data collected at heading-flowering stage. **(B)** Frequency distribution of maximum disease severity (MDS) for 221 F_2:3_ lines grown at Yangling and Tianshui in 2015. **(C)** Frequency distribution of relative area under the disease progress curve (rAUDPC) for 175 F_6_ RILs grown at at Yangling, Jiangyou and Tianshui in 2016. Black arrows indicate the parental line means. Distributions of the polymorphic SNPs in each chromosome by 90K **(D)** and 660K **(F)** SNP arrays and positions of SNPs in chromosome 2B **(E, G)**. Selected SNPs (in the red dotted boxes) were analysed in KASP assays.

The authors apologize for this error and state that this does not change the scientific conclusions of the article in any way. The original article has been updated.

