# Supplementary figures and images for: Saturation Mapping of a Major Effect QTL for Stripe Rust Resistance on Wheat Chromosome 2B in Cultivar Napo 63 Using SNP Genotyping Arrays
Source: Front Plant Sci. 2017 Apr 26;8:653. doi: 10.3389/fpls.2017.00653 (PMC5405077; doi:10.3389/fpls.2017.00653)

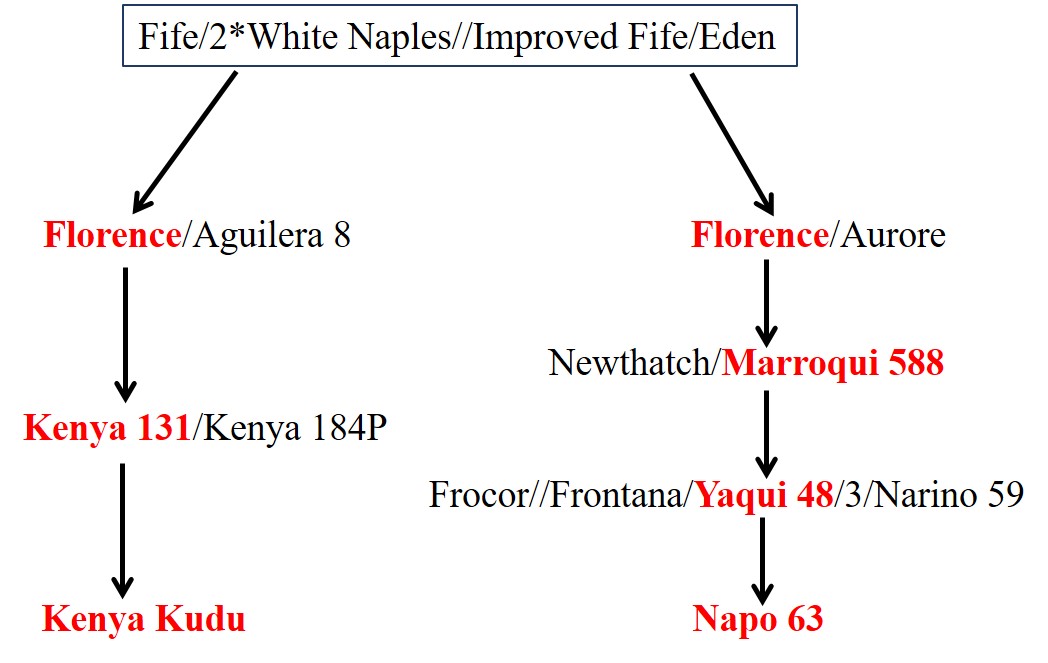

Supplement: FIGURE S1 — Pedigrees of Napo 63 and Kenya Kudu. [file Image_1.JPEG]
